# Retraction: Kim, J.R. and Kim, J.J. Epoxy Resins Toughened with Surface Modified Epoxidized Natural Rubber Fibers by One-Step Electrospinning. *Materials* 2017, *10*, 464, doi:10.3390/ma10050464

**DOI:** 10.3390/ma12081316

**Published:** 2019-04-23

**Authors:** Joo Ran Kim, Jung J. Kim

**Affiliations:** 1 Fiber Science, Cornell University, Ithaca, NY 14853, USA; jk992@cornell.edu; 2 Department of Civil Engineering, Kyungnam University, Changwon-si 51767, Korea

The published article [1] has been retracted at the request of the corresponding author due to a data error in Figure 8, and the citation to article [2] was missed. We note that the retraction of published article [1] was recommended by an investigation carried out by the Cornell University. The paper will therefore be marked as retracted. We apologize for any inconvenience caused by the removal of this article. *Materials* is a member of the Committee on Publication Ethics (COPE) and strives to uphold the highest ethical standards.

## References

[1] Kim J.R., Kim J.J. (2017). Epoxy Resins Toughened with Surface Modified Epoxidized Natural Rubber Fibers by One-Step Electrospinning. Materials.

[2] Kim J.R., Netravali A.N. (2017). One-Step Toughening of Soy Protein Based Green Resin Using Electrospun Epoxidized Natural Rubber Fibers. ACS Sustain. Chem. Eng..

